# Requirement and Development of Hydrogel Micromotors towards Biomedical Applications

**DOI:** 10.34133/2020/7659749

**Published:** 2020-07-10

**Authors:** Xinyi Lin, Borui Xu, Hong Zhu, Jinrun Liu, Alexander Solovev, Yongfeng Mei

**Affiliations:** Department of Materials Science, State Key Laboratory of ASIC and Systems, Fudan University, Shanghai 200433, China

## Abstract

With controllable size, biocompatibility, porosity, injectability, responsivity, diffusion time, reaction, separation, permeation, and release of molecular species, hydrogel microparticles achieve multiple advantages over bulk hydrogels for specific biomedical procedures. Moreover, so far studies mostly concentrate on local responses of hydrogels to chemical and/or external stimuli, which significantly limit the scope of their applications. Tetherless micromotors are autonomous microdevices capable of converting local chemical energy or the energy of external fields into motive forces for self-propelled or externally powered/controlled motion. If hydrogels can be integrated with micromotors, their applicability can be significantly extended and can lead to fully controllable responsive chemomechanical biomicromachines. However, to achieve these challenging goals, biocompatibility, biodegradability, and motive mechanisms of hydrogel micromotors need to be simultaneously integrated. This review summarizes recent achievements in the field of micromotors and hydrogels and proposes next steps required for the development of hydrogel micromotors, which become increasingly important for *in vivo* and *in vitro* bioapplications.

## 1. Introduction

Nano-/micromotors are miniaturized microdevices, which are capable of propelling themselves through solutions by transferring local chemical energy using catalytic reactions or the energy of external fields into autonomous motion [1–7]. The field started from the unexpected discovery of motile Au-Pt bimetallic nanorods self-propelled by self-electrophoresis, i.e., simultaneous oxidation-reduction reactions on Au and Pt nanorod segments in H_2_O_2_ solutions, driven by the reaction 2H_2_O_2_ → 2H_2_O + O_2_ [1, 2]. Since then, nano-/micromotor sizes, shapes, compositions, combinations of materials, and chemical fuels have been optimized to achieve higher chemical-to-mechanical energy conversion efficiencies and realizations of different tasks [7]. During recent years, there has been a growing body of literature investigating biomedical nano-/micromotors [8] for diagnostics and therapy [9–11], operations *in vivo* [12, 13], targeted cargo/drug delivery [14–16], isolation of cancer cells [17], targeting of 3D bladder cancer spheroids [18], micromotor-based immunoassays [19], and integration with cellphone diagnostics [20], among others. Several recent procedures of micromotors' fabrication make noteworthy contributions to simplify the process and add more biocompatible functionalities [21, 22].

However, today considerable challenges exist in applications of nano-/micromotors in real clinical cases [23]. The research on nano-/micromotors, especially catalytic nano-/micromotors, often does not address biocompatibility, biodegradability of materials, and reactions used. Such nano-/micromotors can potentially accumulate in biological tissue and/or release undesired reaction products, and/or they can be rejected by the immune system. Therefore, biocompatible materials and suitable propulsion methods are required for biomedical nano/-micromotors. Moreover, a complex bioenvironment calls for more motion control methods, which need quick and precise responsiveness to certain body signals (e.g.: pH, temperature, and biomolecules). Comprehensive biomedical tasks require task-fulfilling functionalities of nano-/micromotors. Different functionalities to achieve biomedical tasks rely on corresponding biocompatible and functional materials. All these requirements for nano-/micromotors to work *in vivo* thus no doubt add difficulties to the design and production of nano-/micromotors. Among these difficulties, the core is to select and use biocompatible functional materials properly.

The nano-/micromotor field is now increasingly focused on a biocompatible material for *in vivo* applications—the hydrogel. Hydrogels consist of cross-linked polymer networks which are capable of containing a large amount of water with little deformation. Tailored physicochemical properties and similarities to biological tissue enable hydrogels to find broad bioapplications in cardiology, immunology, oncology, tissue engineering, wound healing, on-demand release of drugs, minimally invasive delivery, anticancer treatment, and related biomedical purposes [24–26]. Importantly, a stimuli-responsive polymeric mesh of hydrogels possesses selective permeability to certain molecular species classified by polarity, charge, and size. Stimuli-responsive hydrogels can swell and, therefore, change the size of pores using chemical compositions (e.g., pH) and external triggers (e.g., light and temperature) [27]. An example is hydrophobic poly(methylacrylate) hydrogels that consist of a hydrophobic dense skin and enclosed water for controllable evaporation of water in contrast to conventional hydrophilic polymers [28].

However, these intrinsic properties of hydrogels can help to achieve only local responses, while motile biosystems at the microscale are able to harness the advantages of dynamic motion and swimming over long distances. Recently developed catalytic and externally powered nano-/micromotors are inspired by motile cells and self-propelled motor proteins, which attracted considerable attention to develop man-made multiscale biomedical micromachines [29].

It is noteworthy to mention that the field of hydrogels is currently rapidly evolving from locally responsive and fixed “on-chip” actuators [30, 31] to tetherless bioinspired nano-/micromotors [32, 33]. When integrated with nano-/micromotors, hydrogels will be able to self-propel autonomously, and they can be positioned very precisely and perform targeted delivery of cargo or mechanized minimally invasive operations. As a result, hydrogel micromotors can combine both functions of tetherless micromotors and hydrogels responsive to physical, chemical, and biological triggers. Selective criteria for biomedical hydrogel micromotors are the intersection of the following topics: integration of biocompatible and biodegradable materials; applications of nontoxic reactions and reaction products; and utilization of efficient, motive mechanisms, while keeping the versatile properties of stimuli-responsive hydrogels including full control over molecular diffusion, separation, reaction, and delivery of cells, drugs, or other relevant biomedical cargo.

In this review, the basic requirement and recent development of hydrogel micromotors (sizes in the range of 1-1000 *μ*m) for *in vivo* use are discussed from the perspectives of different propulsion modes, motion control methods, and multiscale biomedical tasks. Hydrogels combined with micromotors can extend short-range dynamic responses while keeping the multifunctional properties of hydrogels for potential operations *in vivo*. Hydrogels' properties, including biocompatibility, biodegradability, superior water absorbent ability, easy modification, and environmental sensitivity, provide alternative routes for micromotors to tackle challenges like biosignal responsibilities and precise drug-releasing control *in vivo*. In the end, an outlook of future challenges and directions are provided for hydrogel micromotors.

## 2. Fabrication Methods and Basic Requirement for Hydrogel Micromotors towards *In Vivo* Applications

During the past years, considerable progress has been achieved in the fabrication and research of micromotors for diverse tasks and in combination with hydrogels. In this section, we briefly introduce basic fabrication methods of hydrogel micromotors. And then we put forward the basic requirement for hydrogel micromotors to be applied *in vivo*: biocompatible propulsion modes, precise motion control methods against a complex bioenvironment, and the ability to accomplish biotasks with minimal invasion/harm to the living body.

### 2.1. Fabrication Methods of Hydrogel Micromotors

Hydrogels can contain cells and drugs, which are protected from the biological environment for programmable release by the diffusion or a targeted delivery of cargo. Subsequently, hydrogel microparticles can be located *in vivo* using intra-articular, intratissue, gastrointestinal, pulmonary delivery, and defect-filling methods. Furthermore, microparticle-based hydrogels help to overcome several limitations of bulk hydrogels, such as a lack of injectability and limited porosity. The large diffusion length of bulk hydrogels limit supplies of nutrients and oxygen to encapsulated cells. The superior properties of hydrogel microcapsules lead to controllable reaction, diffusion, separation, and delivery of nanoparticles and molecular species by a response to stimulus.

To apply microparticle-based hydrogels while getting rid of its local limitation, a micromotor segment is suggested to be combined with the hydrogels. A nano/-micromotor part can be positioned inside or outside the hydrogel particles to fabricate hydrogel micromotors. Generally speaking, micromotors can be produced by a rich variety of fabrication methods including the Layer-by-Layer (LbL) assembly [34], the rolled-up nanomembranes [35], the emulsion solvent evaporation for generation of oil-in-water droplets [36], the glass capillary-based microfluidic lithography [37], the template-assisted electrodeposition [38], the replica molding of polydimethylsiloxane (PDMS) template [39], and the dynamic shadow growth method [40], to name a few examples.

**Figure 1:**
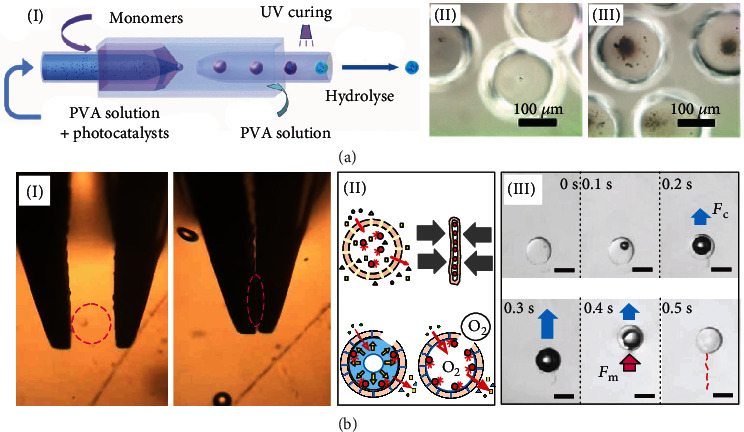
Fabrication of hydrogel micromotors using microfluidics. (a) Hydrogel microparticles with an aqueous core containing photocatalytic nanoparticles. Schematic illustration (I) and optical images of ZnO nanoparticles (II) and TiO_2_ nanoparticles (III) inside the capsule. Reproduced with permission from ref. [42], RSC 2020. (b) Hydrogel micromotors are fabricated using glass capillary-based microfluidics. Mechanical activation of hydrogel microcapsules by compression using metallic tweezers leaves holes/defects in the membrane of hydrogels (I). A schematic image of a hydrogel microcapsule containing catalytic nanoparticles in an aqueous core is shown. The process leads to a membrane rapture and nucleation/generation of an oxygen microbubble during the decomposition of hydrogen peroxide (II). Optical microscopy image sequences of an individual self-propelled microcapsule-based hydrogel micromotor (III). The force produced by a lateral capillary (*F*_*c*_) is indicated using blue arrows, while the force (*F*_*m*_) produced by the catalytic effect is indicated using the red arrow. The two forces are the main motive mechanisms of the hydrogel micromotor. The scale bar is 200 *μ*m. Image is reproduced with permission from ref. [43], IOP 2019.

However, not all fabrication methods of micromotors are suitable for making hydrogel micromotors. Stimuli-responsive hydrogels with a desired nanoscale mesh size can be prepared using standard protocols and high-throughput methodologies, such as batch emulsion, microfluidics, photolithography, electrohydrodynamic spraying, and mechanical fragmentation, followed by an addition of rigid or soft motive power, e.g., magnetic and catalytic micromotor segments. For instance, thermoresponsive hydrogel micromotors were prepared using the oil/water emulsion template method [32]. Such micromotors self-propel at a speed of around 14.8 mm s^−1^ in a 60°C aqueous solution using gas bubbles originating from the thermotropic phase transition. Temperature and pH responsive hydrogels can be made of monomer solutions such as N-isopropylacrylamide (NIPAM) and sodium acrylate or allyl amine, which can be shaped into microtubes using a coaxial flow microfluidic device, followed by hydrogel photopolymerization [41]. Except for its tubular shape, the microfluidic technique can also help to produce thin shell microcapsules with hydrogel shells, and its aqueous core can contain drugs or cells as a cargo. As shown in Figure 1(a), hydrogel microcapsules are fabricated using complex water-in-oil-in-water double emulsion drops generated by the glass capillary-based microfluidics [42]. Thin shell hydrogels with aqueous cores enable faster diffusion of molecular species (e.g., ZnO or TiO_2_ nanoparticles in Figure 1(a)II and III) in comparison to bulk hydrogels [42]. Microfluidics is applied to fabricate microcapsule-based hydrogel micromotors (Figure 1(b)) [43]. In the latter case, catalytic nanoparticles are injected in the aqueous core during the formation of hydrogel microcapsules consisting of methacrylic anhydride. Poly(methacrylic acid) hydrogels are photopolymerized and hydrolyzed to form pH-responsive thin hydrogel shells with controllable permeability. When placed in solutions containing hydrogen peroxide, the microcapsule-based hydrogel micromotors showed distinct promise as novel material for controllable diffusion, reaction, separation, and encapsulation of catalytic nanoparticles and molecular species [43]. Figure 1(b)I–III shows the mechanical activation of hydrogel microcapsules. Mechanical activation of capsule-based hydrogel micromotors can initiate their motion and release of cargo. Microcapsules with catalytic Pt nanoparticles in the core do not generate oxygen microbubbles in a hydrogen peroxide solution due to the small size pores(sub-100 nm) of their networks [43, 44]. However, micro capsules, which are compressed by the metallic tweezers, contain microdefects in the hydrogel membrane, and they activate growth and ejection of oxygen microbubbles from the aqueous catalytic core [43].

Recently, microfluidic methods also developed one-step fabrication of trimethylolpropane ethoxylate triacrylate- (ETPTA-) based micromotors by assembling catalytic nanoparticles (micromotor's segments) onto the surface of ETPTA particles [45, 46], which could be a guide for future one-step fabrication of hydrogel micromotors using droplet microfluidics.

### 2.2. Suitable Propulsion Modes, Motion Control, and Mission Capability *In Vivo*

If hydrogel microparticles contain rigid/soft micromotor segments, questions related to material biocompatibility, fuel reactions to toxicity, and immune system response to hydrogel micromotors should be studied. Various materials have been considered for biomicromotors, such as polymers, metals, inorganic oxides/salts, and biohybrids [47]. For instance, noncytotoxic polymers, metals (e.g., Zn, Mg, and Al), inorganic oxides (e.g., SiO_2_, CaCO_3_, GO, TiO_2_, and MnO_2_), and certain biohybrids (e.g., proteins, enzymes, vesicles, and plants) have good biocompatibility or controllable biodegradability for micromotor operations *in vivo*. To avoid accumulation in tissue/organs, micromotors can degrade, dissolve, or “self-erase” in time, after the task has been accomplished. Moreover, since catalytic micromotors are powered by catalytic reactions, another challenge concerns finding biocompatible chemical fuels and reaction products for *in vivo* operations. One potential solution is the utilization of fuels and reaction products which are parts of metabolic pathways, such as an application of CO_2_ gaseous fuel [48].

**Figure 2:**
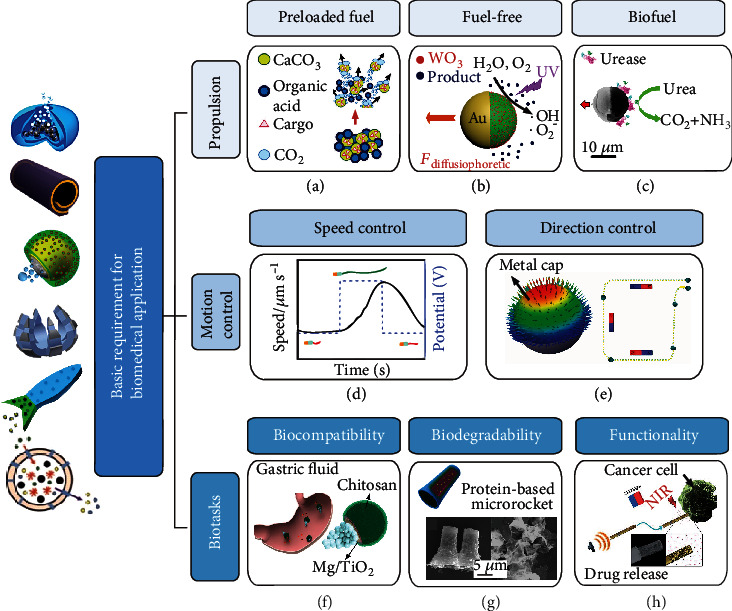
Strategies developed by versatile autonomous and externally powered/controlled micromotors to satisfy requirements for bioapplications. (a) “Preloaded fuel”: reproduced with permission from ref. [54], AAAS 2015. (b) “Fuel-free”: reproduced with permission from ref. [57], American Chemical Society 2017. (c) “Biocompatible fuel”: reproduced with permission from ref. [67], American Chemical Society 2016. (d) “Speed control”: reproduced with permission from ref. [69], The Royal Society of Chemistry 2009. (e) “Direction control”: reproduced with permission from ref. [70], American Chemical Society 2012. (f) “Biocompatibility”: reproduced with permission from ref. [16], Springer 2017. (g) “Biodegradability”: reproduced with permission from ref. [75], American Chemical Society 2015. (h) “Functionalization”: reproduced with permission from ref. [76], Wiley-VCH 2014. Left-side schematics of different architectures of micromotors (from top to bottom): self-propelled supramolecular motor with temperature-responsive velocity regulation, reproduced with permission from ref. [65], Springer 2017; flexible thermoresponsive self-folding polymeric-Pt tubular micromotor, reproduced with permission from ref. [80], Wiley-VCH 2014; Mg/Pt-PNIPAM Janus micromotor applied for temperature-controlled release of drugs using a surface-attached thermoresponsive PNIPAM hydrogel, reproduced with permission from ref. [14], American Chemical Society 2014. Thermoresponsive therapeutic theragrippers consist of rigid PPF segments and stimuli-responsive PNIPAM-AAc hinges, reproduced with permission from ref. [81], Wiley-VCH 2014. A fish-like enzymeless micromotor composed of a phenylboronic acid modified PNIPAM hydrogel and a SDS surfactant, reproduced with permission from ref. [82], The Royal Society of Chemistry 2017. The microcapsule-based micromotor with a catalytic liquid core and a hydrogel shell is reproduced with permission from ref. [43], IOP 2019.

Here, we discuss basic requirements to apply hydrogel micromotors *in vivo* from three perspectives: biocompatible propulsion modes, motion control ability, and task-fulfilling capability. These requirements are also suitable for other biomedical micromotors. Although not all examples of micromotors in Figure 2 could really perform biomedical tasks, they meet a few conditions for biomedical applications and thus together help to understand the total needs for micromotors to work *in vivo*.

#### 2.2.1. Biocompatible Propulsion Mode

Although H_2_O_2_ is a widely used fuel molecule to power catalytic micromotors [36, 40, 49–51], the peroxide fuel is not suitable for *in vivo* uses due to its cytotoxicity [52]. Recent studies of biomicromachines have heightened the need for finding novel biodegradable and biocompatible materials and reactions and efficient biocompatible propulsion mechanisms for motion at the microscale. One strategy can be realized by the preloading of solid/liquid fuels directly “on-the-board” of micromotors, which are however consumed during motion [14, 53]. For instance, the CaCO_3_ micromotor can use CaCO_3_ as solid fuel to react with acid, releasing CO_2_ bubbles (Figure 2(a)). The micromotor can then be propelled by CO_2_ bubbles and deliver protein cargo through wounds and into the vasculature to halt the hemorrhage [54]. Recent demonstrations show fuel-free micromotors powered by external fields. For instance, applications of magnetic field [55, 56], light source [57–62], ultrasound [63, 64], temperature [65], and electric field [66] are used to generate the motive force of externally powered/controlled micromotors. For example, a light-powered Au-WO_3_@C motor, shown in Figure 2(b), consists of a colloidal carbon WO_3_ nanoparticle composite. The motor is able to move in aqueous solution and perform photodegradation of organics at rates faster than diffusion [57]. Using biocompatible fuel is an alternative propulsion method. For example, hollow silica mesoporous Janus spheres have been demonstrated to self-propel in glucose and urea solutions (Figure 2(c)) [47, 67].

#### 2.2.2. Motion Control Capability against Complicated Biomedical Environments

The second requirement for nano-/micromotors concerns a control of their motion. The goal here would be to utilize micromotors for navigation in the smallest capillaries (i.e., below 10 *μ*m) and achieve precise positioning for a stimuli-responsive release of molecules or mechanical operations [68]. The motion control strategies include speed control and direction control. If chemical fuels and catalytic segments are used, micromotors change their speeds by altering the fuel concentration, the catalyst turnover rate, and the surface area of catalysts. One example in Figure 2(d) shows the “on-off” control of the speed of catalytic nanomotors realized by the modulation of electrochemical potential [69]. In another example, motion direction of micromotors can be controlled by changing the direction of a magnetic field when the micromotors are equipped with magnetic nano/-microstructures. Self-propelled Janus micromotors with catalytic/magnetic caps are controlled by an applied magnetic field for the on-chip delivery of cargo [70]. In addition, the advantages of the micromotor shape of ultraprecise motion control in three-dimensions can be explored. Micromotors with different 3D configurations exhibit distinct motion behaviors and have been applied to different scenarios. Taking the micromotors controlled by magnetic field as examples, a magnetic helical microstructure powered by rotating magnetic fields can show rotation or corkscrew motion [37]. This kind of helical micromotor, when equipped with a hard shell, is particularly suitable to remove necrotic tissue because the corkscrew motion and the helical shape together could make the micromotor work as a microdrill. In another example, a micromotor with a spherical shape is attractive for targeted drug delivery because of the potentially easy fabrication and large volume to contain drugs. Janus micromotors with a magnetic multilayer cap are capable of doing deterministic motion at a large scale in an external magnetic field. This directional motion enables Janus micromotors to deliver cargos precisely [70]. In one word, changing the configurations of micromotors is not only a strategy to achieve speed/direction control but is also a smart way to better fulfill various biotasks relying on different advantages of 3D configurations.

Ultimately, externally controlled micromachines can potentially navigate in arteries and capillaries, and novel methods need to be developed to monitor real-time positions of nano-/microdevices deep in the tissue. If combined with real-time optoacoustic tracking [71] or magnetic resonance microscopy [72], it is feasible to observe individual microobjects. Recently, multifunctional microbubbles with engineered shells and a gaseous core, fabricated using microfluidic methods, are particularly attractive for biomedical ultrasound imaging and theranostics [73, 74].

#### 2.2.3. Task-Fulfilling Capability

For micromotors really functionalizing *in vivo*, two prerequisites are needed to be fulfilled: biocompatibility and biodegradability (or motor retrievability). Biocompatibility ensures that micromotors would not harm a tissue or be attacked by the immune system. For example, biocompatible micromotors can consist of magnesium, titanium dioxide, and chitosan, which would contribute to an active drug delivery, e.g., to the mouse stomach [16], as shown in Figure 2(f). Biodegradability of micromotors means that the micromotors are capable of “self-erasing” or dissolving in time [14, 53]. During biodegradability, micromotors can be broken down into simpler substances (carbon dioxide, water, methane, etc.) by enzymes. Figure 2(g) shows an example of a micromotor consisting of biodegradable bovine serum albumin/poly-l-lysine that is biodegradable and can be used for anticancer drug delivery triggered by infrared light [75]. Last but not the least, micromotors need to accomplish a specific “mission” like drug delivery *in vivo*. As shown in Figure 2(h), a nanoporous Au wire motor powered by ultrasound can release drugs as designed [76]. Other important tasks of biomedical nano-/micromotors include self-propelled nano-/microtools and minimally invasive surgery [77]. For instance, sharp magnetic [78] and autonomous chemically driven tubular nano-/microtools [79] have been demonstrated for drilling operations of cell material and animal liver, correspondingly.

## 3. Recent Developments of Hydrogel Micromotors

Hydrogel micromotors with novel architectures and functions can provide controllable permeability, tunable release kinetics, encapsulation of chemical reactions, separation, and on-demand delivery of cargo payloads [24–32, 41, 83]. Recently, multiple studies have been focused on novel designs and combinations of hydrogels and micromotors [65, 82, 84]. This section discusses how hydrogel micromotors function with the help of the hydrogel part to achieve different means of propulsion, motion control, and tasks.

### 3.1. Propulsion Strategies of Hydrogel Micromotors

Hydrogel hybrids with incorporated nano/-microstructures physically or chemically linked to the hydrogel network have been well recognized, and a number of advantages have been already explored including enhanced hydrogel stiffness, biocompatibility of hydrogel surface, desired viscoelasticity, and an ability to carry and protect embedded nano/-microstructures (e.g., polymeric, carbon based, and metallic) [85]. In the latter case, stable hydrogels can be prepared by the encapsulation of nano/-micromotors, followed by a subsequent stabilization of the hydrogel matrix by chemical photopolymerization or enzymatically cross-linking methods. However, bulk hydrogels are not always suitable for specific applications. For example, in certain cases (i) a precise injection of hydrogel particles with sizes in the range 1–1000 *μ*m in tissue/organ is required or (ii) a full spatiotemporal control over release of drugs can be achieved by particle diameter, hydrogel mesh, and molecular interactions of hydrogel and drugs [86]. Larger particles (more than 100 *μ*m in diameter) are highly influenced by gravity that leads to particles' rapid sedimentation in aqueous media, and therefore, smaller sizes for hydrogel micromotors are required.

**Figure 3:**
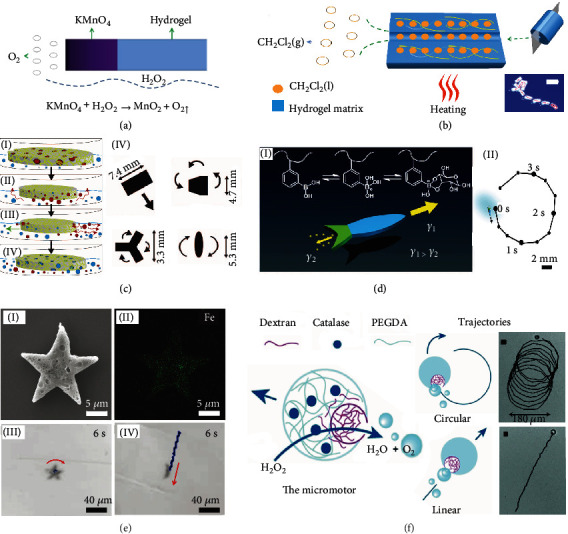
Different propulsion strategies of hydrogel micromotors. (a) Bubble-propelled hydrogel micromotor including a catalyst (KMnO_4_). Reproduced with permission from ref. [84], American Chemical Society 2016. (b) Bubble-propelled emulsion-based hydrogel micromotor due to absorbing fuel (CH_2_Cl_2_ liquid). The inset shows a typical trajectory of an emulsion-based hydrogel micromotor (scale bar 20 mm). Reproduced with permission from ref. [32], American Chemical Society 2017. (c) Schematics (I)~(IV) illustrate an organic solvent-driven motion of an oval-shaped PNIPAM gel. Dark red circles represent ethanol, and the light blue circles are water. Different movements are observed with different shapes of hydrogel micromotors. Reproduced with permission from ref. [87], American Chemical Society 2008. (d) The autonomous movement of a fish-like enzymeless motor, caused by the Marangoni effect, which appears due to an interaction between the glucose and the hydrogel with phenylboronic acid (I); the corresponding trajectory of a hydrogel micromotor is shown (II). Reproduced with permission from ref. [82], The Royal Society of Chemistry 2017. (e) Magnetic propulsion of a star-like hydrogel microswimmer by including ferromagnetic Fe_3_O_4_ nanoparticles in the hydrogel. Reproduced with permission from ref. [55], Wiley-VCH 2018. (f) Bubble propulsion of the asymmetric hydrogel motor by the catalase contained in the hydrogel matrix, which converts hydrogen peroxide into water and oxygen. The trajectory of the motor is either circular or linear, depending on the direction of bubble recoil (scale bar 20 *μ*m). Reproduced with permission from ref. [89], Wiley-VCH 2018.

Recent demonstrations of hydrogel micromotors include both hydrogel microparticles with embedded and externally connected nano/-microstructure motor segments. Hydrogel micromotors with different propulsion strategies are shown in Figure 3. Using the large volume inside hydrogels to preload fuels or catalysts is one hydrogel-assisted propulsion method. Like absorbing water, hydrogels can absorb fuels and trap catalysts in liquid or solid states. Wang et al. [84] demonstrated a catalyst preloading method (Figure 3(a)) by injecting saturated KMnO_4_ solution to form segmented cylindrical hydrogel micromotors. When the hydrogel micromotors self-propel in H_2_O_2_ fuel, KMnO_4_ and its product MnO_2_ catalyze H_2_O_2_ and generate O_2_ bubbles. The same group demonstrated fuel loaded “on the board” of hydrogel micromotors [32] (Figure 3(b)): a low-boiling-point CH_2_Cl_2_ oil is loaded in the hydrogel matrix. Subsequently, under thermal stimulus, the hydrogel micromotors are propelled by the CH_2_Cl_2_ bubbles. In these experiments, outstanding properties to absorb, permeate, and release small molecules using acrylamide hydrogels ensure the retention of the KMnO_4_/CH_2_Cl_2_ solution and the release of O_2_/CH_2_Cl_2_ bubbles.

Another interesting methodology has been employed using SDS/PA-co-PN hydrogel micromotors powered by glucose fuel and the Marangoni effect [33]. Upon the addition of glucose, the 3-acrylamidophenylboronic acid (AAPBA) layer shifts the equilibrium between the charged and uncharged states in the hydrogel. This effect leads to an appearance of hydrophilic regions, which releases sodium dodecyl sulfate (SDS) and increases the swelling of the hydrogel. The Marangoni effect, based on the mass transfer along an interface between two fluids due to a built-in gradient of the surface tension, is used as an effective motive mechanism for hydrogel micromotors [6]. The microporous structure containing a solvent/solution can diffuse out of the hydrogel surface and interact with the bulk liquid.  Figure 3(c) [87] shows various hydrogel structures consisting of poly(N-isopropylacrylamide) (PNIPAM), which are presoaked in ethanol. When placed in water, the structures self-propel autonomously by the Marangoni effect. Shapes of micromotors lead to different trajectories of motion, including linear, rotary, and helical trajectories due to a unique balance of drag and motive forces.  Figure 3(d) shows a fish-like hydrogel micromotor, where a phenylboronic acid-modified PNIPAM hydrogel (preloading with a surfactant) self-propels autonomously using glucose as a fuel [82]. An interaction between the glucose and the phenylboronic acid changes the swelling behavior of PNIPAM to extrude a surfactant, thus propelling the motor using the Marangoni effect.

The cross-linked hydrogels can also trap functional nanoparticles (e.g., magnetic nanoparticles) or biomolecular catalysts, helping propulsion by external fields/fuels. Applications of external fields, e.g., a magnetic field and a light source, are other examples to power actuators with muscle-like flexibility [88]. For example, star-like poly(vinylalcohol)/alginate biodegradable hydrogel micromotors incorporate magnetic nanoparticles by gelation (Figure 3(e)); the structures are subsequently actuated in a rotating magnetic field [55]. Further advantages of nanoparticle-hydrogel composites have been explored in soft actuators, clinical implants, and environmental remediation using catalytic oxidation of toxins (Figure 3(f)) [89]. The micromotor consists of a poly(ethylene glycol) diacrylate (PEGDA) hydrogel matrix and dextran with catalase trapped in PEGDA. The catalase decomposes the H_2_O_2_ fuel, leading to generation of O_2_ bubbles. The biocompatible PEGDA hydrogel contains active catalase and an efficient concave microcavity, which typically reduces the energy barrier for bubble nucleation by diffusion (in contrast to convex and flat surfaces) [89].

In addition, biohybrid micromotors, which integrate biological cells with synthetic components, are opening a new way of biocompatible propulsion by using cells (e.g., bacterial cells) as a micromotor segment. Biocompatible and biodegradable hydrogel materials like poly(ethylene glycol) (PEG), with low toxicity and low immunogenicity, are the focus in cargo materials for drug delivery micromotor systems with bacterial cells [90]. For example, motile bacteria are immobilized onto bacterial cellulose structures, forming a microbial biohybrid motor with an average speed of 4.8 *μ*m/s. The bacterial cellulose, a kind of hydrogel, is verified as having greater affinity for motile bacteria than other polymers like PDMS [91].

### 3.2. Motion Control of Hydrogel Micromotors Using Local Chemical and External Triggers

Stimuli-sensitive hydrogels (smart hydrogels) in hydrogel micromotors could be designed to regulate the micromotors' motions according to their “smart” response to subtle environment changes. Stimuli-responsive hydrogels, i.e., hydrogels induced by light, thermal, and pH triggers of polymers, are particularly attractive in textile transdermal drug delivery and tissue engineering applications. These smart hydrogels experience swelling and porosity change, and due to mechanical elasticity, hydrogels revert and shrink to their original shape/porosity after stimuli are removed. The cationic pH-responsive hydrogels, such as chitosan, poly(N,N-dimethylaminoethyl methacrylate) (PDMAEMA), and poly(N,N-diethylaminoethyl methacrylate), increase polymer matrix porosity in acidic pH and decrease the porosity in basic pH solutions, while pH-responsive polymers such as albumin and polyacrylic acid increase porosity in basic pH and shrink in acidic pH [92]. Hydrogels experience charge density redistribution in functional pendant groups due to variations in pH, which lead to electrostatic repulsion between adjacent chains that induces water adsorption. Both natural (e.g., chitosan, cellulose, albumin, and gelatin) and synthetic (e.g., pluronic F127, PEG, PDMAEMA, and PNIPAM) polymers can be applied for a responsive delivery of molecular species for anticancer drug delivery, antibacterial treatments, skincare procedures, and tissue engineering. Considering their optimal adaptability, rigidity, and softness, stimuli-responsive hydrogel actuators can respond to environmental cues: deform, bend, swell/shrink, and move by light, pH, temperature, redox reactions, humidity, and electrical and magnetic fields [93]. The mechanism of temperature-responsive (e.g., PNIPAM) hydrogels is based on temperature-dependent swelling behavior in an aqueous phase, particularly of high interest with critical solution points near physiological temperature to initiate delivery of drugs. On the other hand, the electric response (e.g., in chitosan and PAAc) is caused by properties of hydrogel electrolytes, e.g., electrophoretic, Coulomb, and electroosmotic interactions, where cations move to a cathode and anions move to an anode under an applied electric field. This effect causes changes in the osmotic pressure, expansion/swelling, and shrinking/deswelling of hydrogels.

**Figure 4:**
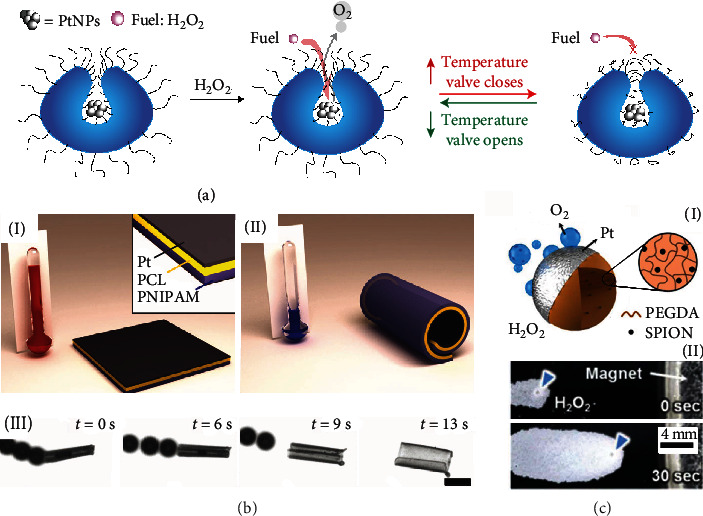
Various motion control methods of hydrogel micromotors. (a) Velocity control of PNIPAM-modified stomatocyte hydrogel micromotors using thermosensitive PNIPAM hydrogel. Reproduced with permission from ref. [65], Springer 2017. (b) Activation of PNIPAM-Pt hydrogel micromotors by bubbles during reversible rolling of layers at different temperatures (I)~(II) (scale bars: 50 *μ*m). Reproduced with permission from ref. [80], Wiley-VCH 2014. (c) Direction control by enclosing SPION magnetic nanoparticles in the hydrogel matrix and in a magnetic field. Reproduced with permission from ref. [72], EU 2015.

Today, the first generation of man-made nano-/micromachines, consisting of self-propelled and externally powered microparticles, can perform a number of tasks such as isolation of pathogens and targeted delivery of cargo. First steps have been made to control speeds and directionalities of individual and multiple tetherless micromotors. Responsive hydrogel micromotors can propel towards the target of interest and react to changes of the local chemical environment, while simultaneously absorbing or releasing molecular species.  Figure 4(a) shows an example of velocity modulation of stomatocyte micromotors using a temperature-sensitive PNIPAM hydrogel brush [65]. The swelling behavior of a PNIPAM hydrogel changes with the temperature altered above or below the lower critical solution temperature (LCST) of PNIPAM. The opening of the stomatocyte micromotor is regulated by the PNIPAM hydrogel brush and thus it controls the access of H_2_O_2_ fuel to catalytic Pt nanoparticles. Another similar example is speed regulation of hydrogel micromotors by light-sensitive hydrogel valves. This example shows a biomimetic bottom-up supramolecular approach to assemble micromotors, i.e., block the copolymers forming stomatocytes with Pt nanoparticles and modulate speed using blue-light-responsive valves, which regulate the diffusion of hydrogen peroxide fuel [94, 95].  Figure 4(b) illustrates a PNIPAM multilayer hydrogel micromotor [80]. The micromotor consists of the Pt layer, the polycaprolactone (PCL) layer, and the PNIPAM layer. The hydrogel micromotor reversibly folds and unfolds at different temperatures. The micromotor with a smaller tubular diameter ejects O_2_ microbubbles at higher frequencies during the decomposition of H_2_O_2_. By changing the 3D configuration of micromotors, speed regulation is achieved. This observation is in good agreement with previous results, indicating that a higher aspect ratio of catalytic microtubes, i.e., length/diameter ratio, leads to a more efficient confinement of molecular species driven by diffusion. Subsequently, a supersaturation of gas molecules in microtubes with a high aspect ratio leads to a lower threshold concentration for heterogeneous nucleation of gas microbubbles [80].

Except for stimuli-responsive hydrogels, other conventional hydrogels could also help to control the motion of micromotors. This motion control method requires the incorporation of functional nano-/microparticles or nano-/microstructures (e.g., Co, Ni, and Fe_3_O_4_) into the hydrogel matrix or magnetic coatings on the surface. This enables “fuel-free” tetherless motion control with high precision. External fields can influence velocity and direct trajectories of motion.  Figure 4(c) shows an example of the direction control of an individual PEGDA-based hydrogel micromotor containing superparamagnetic iron oxide nanoparticles (SPION; Fe_3_O_4_) [72]. To name a few more, a biomimetic hydrogel microrobot, containing aligned iron oxide nanoparticle chains embedded in the microgel shell, achieves accurate positioning control in a collective manner, which is promising for future biomedical applications in a complex environment [96].

### 3.3. Task-Fulfilling Capability of Hydrogel Micromotors

**Figure 5:**
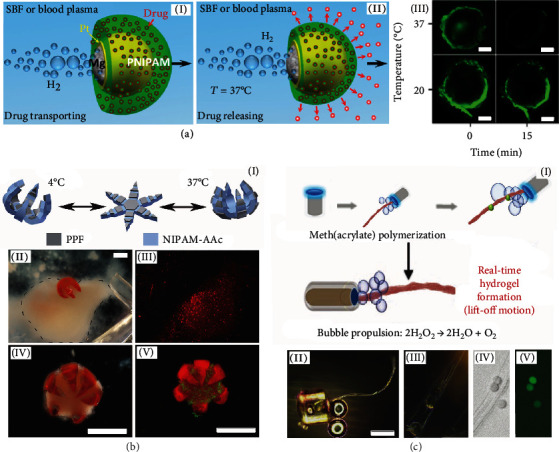
Biomedical chemomechanical responsive hydrogel micromotors for cargo delivery and related potential biomedical operations. (a) Transportation (I) and delivery of drugs (II) by the Mg/Pt-PNIPAM Janus hydrogel micromotors using temperature modulation; (III) fluorescent image indicating the release of drugs from the Mg/Pt-PNIPAM hydrogel micromotor (scale bars, 10 *μ*m). Reproduced with permission from ref. [14], Copyright 2014, American Chemical Society. (b) Drug-releasing theragrippers, which capture tissue and deliver drugs activated by a change of temperature and mechanical strain between the PNIPAM-AAc hinges (scale bars, 1 mm). Reproduced with permission from ref. [81], Copyright 2014, Wiley-VCH. (c) TRAP hydrogel micromotor activation (I) powered by the real-time polymerization of a photocurable hydrogel with fibroblast cells (II~V) (scale bar, 200 *μ*m); (IV) bright field image of an individual hydrogel micromotor; (V) fluorescent image of living cells (wavelength 517 nm). Reproduced with permission from ref. [98], Copyright 2019, Wiley-VCH.

Stimuli-responsive soft hydrogels actuated by electric and magnetic fields, light, temperature, pH, and humidity have been already demonstrated for their motion control strategies. These smart hydrogels are able to work further in hydrogel micromotors to accomplish biomedical tasks: controllable drug delivery, nano-/microsurgeries, and so on.  Figure 5 illustrates examples of hydrogel micromotors applied for delivery and mechanical operations. Responsive hydrogel micromotors are programmed to deliver and release drugs on demand [97]. The responsiveness of chemically/physically sensitive hydrogels can change a hydrogel mesh at the nanoscale due to the hydrogel swelling property and the delivery of drug molecules at a controllable rate. For example, the PNIPAM hydrogel on Mg/Pt-PNIPAM Janus micromotors can load, transport, and release drugs by temperature change, as shown in Figure 5(a) [14]. Similar cases include Janus magnetic PNIPAM hydrogel micromotors that respond to temperature changes and adsorb erythromycin in water [51]. The water absorbent property of hydrogels enables the uptake of hydrophilic drugs, while the swelling property that is altered by temperature assures the release of drugs. For instance, when temperature increases above LCST (~37°C), PNIPAM hydrogel's network shrinks and releases drug molecules. When chemically sensitive hydrogels respond to change in pH, hydrogels can elongate and create a bending momentum between layers. This approach has been applied to assemble responsive micro-chemo-mechanical structures. One example is shown in Figure 5(b): theragrippers consisting of a rigid poly(propylene fumarate) (PPF) layer and smart poly(N-isopropylacrylamide)-co-acrylic acid (PNIPAM-AAc) hydrogel hinges [81]. The swelling behavior of the PNIPAM-AAc hydrogel changes, subsequently the theragrippers capture the tissue and simultaneously release the preloaded drugs. Besides, the microgrippers are capable of capturing and tearing off a part of the biomaterials for a tissue sampling operation, for example. The biocompatibility, softness, and flexibility of hydrogels in integration with rigid and sharp structures lead to a property to perform micromechanical operations [81].

Encapsulating and transporting cells are other bioapplications for biocompatible hydrogels.  Figure 5(c) shows Pt NPs incorporated in PEGDA cylindrical micromotors with one side consisting of PEGDA monomers that are able to polymerize in situ [98]. When the micromotor self-propels in H_2_O_2_ fuel, PEGDA monomers polymerize into a thread-like structure, which can trap cells. Moreover, since nutrients can diffuse in hydrogel thin films, hydrogels are particularly attractive for the encapsulation and delivery of viable cells. Another novel cell encapsulation case is to apply Fe_3_O_4_ nanoparticle-doped alginate hydrogels as a coating layer around microbial cells [99]. This live cell system demonstrated high magnetic responsiveness with an applied magnetic field. Here, hydrogel is not only used to encage cells as an artificial extracellular matrix (ECM) but also to trap and employ functional Fe_3_O_4_ nanoparticles to be micromotor segments.

From the above examples of hydrogel micromotors, different functionalities of hydrogels in the micromotor systems are demonstrated. The biocompatibility of a hydrogel meets the minimum requirement of nano-/micromotor materials for biomedical use. Some hydrogels are biodegradable, which is prefered in biomaterials used to fabricate nano-/micromotors. The cross-linked microporous structure of hydrogel microparticles directly contributes to its soft and tissue-like physical properties, giving hydrogels a liquid/drug/fuel absorbent or preloading ability. The surface of hydrogels is easily modified with functional groups which can recognize certain biomolecules and thus changes swelling behaviors or other physical properties of the hydrogels. This property of facile surface modification has allowed modified hydrogel micromotors to be applied as biosensors such as a biosensor for the detection of glucose [82]. Another property of environment-sensitive hydrogels is their quick response to external/chemical stimuli which has been widely made use for temperature-controlled drug delivery. The development of various environment-sensitive hydrogels with optimal sizes, shapes, propulsion modes, and methods of cargo release for therapeutical uses together with hydrogel micromotors is the next important step. The porosity of hydrogel microparticles makes it possible to trap, release, and deliver cells/biomolecules/functional nanoparticles [100]. Trapping magnetic and catalytic nanoparticles could enable the combinatorial magnetic-catalytic propulsion and motion control of the hydrogel micromotors by chemical fuels and an external magnetic field.

## 4. Future of Hydrogel Micromotors: Experiences from Other Hydrogel Devices

One of the major current challenges of hydrogel micromotors is a reduction of hydrogels to microscale particles and the integration of a versatile motion control for micromotors, for which hydrogel biocompatibility, biodegradability, and task-fulfilling ability are not lost. The field of stimuli-responsive, bioactive, biocompatible, flexible hydrogel microparticles is rapidly evolving and integrating chemically, mechanically, physically, and biologically with potential biomedical procedures for drug delivery, tissue engineering, minimally invasive delivery, and surgery [101, 102]. For future real *in vivo* application of hydrogel micromotors, already developed hydrogel devices, hydrogel microparticles, and hydrogel-based soft robotics are discussed here.

**Figure 6:**
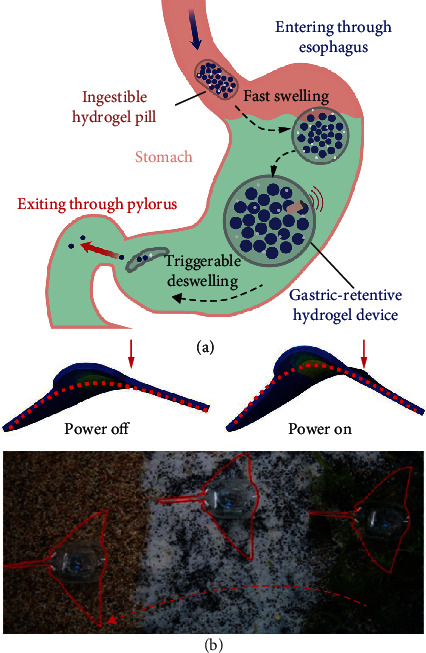
Applications of hydrogel microparticles *in vivo* and an actuation of a soft robot. (a) Schematic illustration of the gastric-retentive hydrogel pill, which swells in the stomach. Reproduced with permission from ref. [103], Springer 2019. (b) Schematic device illustration and optical microscopy image of the hydrogel electronic fish with an integrated power system on-the-board. Reproduced with permission from ref. [104], AAAS 2017.

Hydrogel microparticles have been used in a wide range of drug-delivery systems [86]. For instance, Figure 6(a) shows an ingestible gastric-retentive hydrogel device working *in vivo* as a noninvasive alternative method [103]. For hydrogels to remain in gastric residency, the materials are required to withstand mechanical forces and retain robustness for a long time, which is often difficult to achieve. One solution is a device the size of a pill, which swells and does not fracture under applied forces in the stomach. Another example concerns soft robotics (~10 cm in size), which has more integrated functionality than hydrogel micromotors but larger in size. Figure 6(b) illustrates a fast-moving soft electronic fish with an integrated dielectric elastomer muscle, silicone body, power, and remote control [104]. One electrode represents an encapsulated hydrogel, while the other electrode is the surrounding water. Applied voltages accumulate charges on the dielectric elastomer, and the fish propels by flapping movements. The surface of hydrogels can be chemically modified with functional groups for specific biomolecular interactions for a controllable swelling behavior. Figure 6(b) shows positions of the electronic fish in time with an integrated on-board power. Hydrogel soft robotics can quickly respond to biochemical changes and external stimuli. When comparing hydrogel microparticles, micromotors, and robotics with larger sizes together, hydrogel microparticles are best suited in the bioenvironment with microsizes while unable to move autonomously; hydrogel-based robotics possess the most integrated functionals and precisely controlled motion trajectories often at the cost of biocompatibility and a larger size; hydrogel micromotors, in the middle, take the advantages of their microparticle size and autonomous movements but are less biocompatible than hydrogel microparticles and less functional/power integrated than soft hydrogel robotics. These two examples in Figure 6 reveal the prospects and future of hydrogel micromotors, which combine the mature biocompatible drug-delivery ability of hydrogel microparticles with the nano-/microsize and power-on-board integrated functionalities of hydrogel soft robotics together.

Two fabrication routes are suggested here: one is to equip hydrogel microparticles with more biocompatible/functional micromotor segments and the other one is to downsize hydrogel-based soft robotics. From the perspective of the nano-/micromotor field, the former route is widely adopted by now. Engineering of efficient methods to navigate hydrogel microparticles, with spatiotemporal drug-release profiles, delivery of cells, minimally invasive surgery, and other operations is of high interest for a broad range therapies and precision medicine [105]. Particularly, one important application concerns tumor drug delivery using hydrogel particles, which causes less side effects than conventional chemotherapy. In the latter case, biodegradable polymers can be used (e.g., polyphosphazene, hyaluronic acid, chitosan, polyesters, and alginate) [106]. Specifically, multiscale hydrogel micromotors based on microgels (0.5−10 *μ*m) and nanogels (<200 nm) can be prepared for different delivery routes towards cancer treatment. Such nanogels can be used intravenously, while microgels can be applied in oral, pulmonary, and transarterial deliveries. Using externally controlled hydrogel micromotors can help to accumulate drug carriers in specific locations, which are otherwise inaccessible. At the same time, it helps to reduce the toxic side effects of high therapeutic concentrations. Otherwise, drugs are cleared from the organism in a short time.

## 5. Conclusion and Outlook

This review summarizes basic requirements and recent developments for the integration of hydrogels and micromotors, i.e., hydrogel micromotors, for biomedical applications, providing an abundance of opportunities for both hydrogels and micromotors with unique propulsion mechanisms, motion control methods, and delivery of cargo payloads. Over the last decade, micromotors have made pronounced progress towards biomedical application. However, the main limitations today are biocompatibility of micromotor materials, chemical fuels, and reaction products for *in vivo* uses. Hydrogels and micromotors started as two separate disciplines, and in recent years, a convergence of these previously separated fields occurred, which can lead, in our opinion, to the next generations of smart multiscale multifunctional biomicromachines. For example, local dynamic responses of hydrogels can be extended if integrated with micromotors for a very precise positioning, molecular cargo/cells can be released in the right place, and minimally invasive mechanical operations can be performed. Moreover, hydrogel micromotors can combine hydrogel properties including the absorption of a large amount of water, mechanically flexible/soft structures, bioactivity, biocompatibility, controllable separation, reaction, and diffusion. Micromotors can be powered by different motive mechanisms (self-electrophoresis, self-diffusiophoresis, bubble recoil, Marangoni effect, or external fields), which can be chosen for a specific bioapplication. Recent progress in micromotor research of biocompatible fuels, such as glucose and urea are of particular interest for the development of biocompatible chemical fuels [67].

Although hydrogel micromotors are now toddlers with a rotund tummy, this newly developing field sees a bright future from developed applications of hydrogel microparticles and hydrogel-based soft robotics [101, 102, 107, 108]. If combined with motive power, multifunctional hydrogels at the microscale will play important and diverse roles in biomedical procedures in the near future. (For polymer names that appear at least twice in the text, we present a table in the Abbreviations.)

## Abbreviations

PDMS: Polydimethylsiloxane
AAPBA: 3-Acrylamidophenylboronic acid
SDS: Sodium dodecyl sulfate
NIPAM: N-Isopropylacrylamide
SDS/PA-co-PN: Copolymerization of AAPBA and NIPAM in the presence of SDS
PEG: Poly(ethylene glycol)
PDMAEMA: Poly(dimethylaminoethyl methacrylate)
PAAc: Poly(acrylic acid)
PEGDA: Poly(ethylene glycol)
PPF: Diacrylatepoly(propylene fumarate)
PNIPAM: Poly(N-isopropylacrylamide)
PNIPAM-AAc: Poly(N-isopropylacrylamide)-co-acrylic acid
PCL: Polycaprolactone.
